# Second case of European bat lyssavirus type 2 detected in a Daubenton’s bat in Finland

**DOI:** 10.1186/s13028-017-0331-y

**Published:** 2017-09-25

**Authors:** Tiina Nokireki, Tarja Sironen, Teemu Smura, Veera Karkamo, Liisa Sihvonen, Tuija Gadd

**Affiliations:** 1 Finnish Food Safety Authority Evira, Mustialankatu 3, 00790 Helsinki, Finland; 2 Department of Virology, University of Helsinki, P.O. Box 21, 00014 Helsinki, Finland; 3 Department of Veterinary Biosciences, Faculty of Veterinary Medicine, University of Helsinki, P.O. Box 66, 00014 Helsinki, Finland

**Keywords:** Daubenton’s bat, EBLV-2, European bat lyssavirus type-2, Finland, *Myotis daubentonii*

## Abstract

European bat lyssavirus type 2 (EBLV-2) was detected in Finland in a Daubenton’s bat (*Myotis daubentonii*) found in the municipality of Inkoo (60°02′45″N, 024°00′20″E). The bat showed neurological signs and was later found dead. The laboratory analysis revealed the presence of lyssavirus, and the virus was characterized as EBLV-2. This isolation of EBLV-2 was the second time that the virus has been detected in a Daubenton’s bat in Finland. This provides additional proof that EBLV-2 is endemic in the Finnish Daubenton’s bat population.

## Findings

Rabies is a fatal encephalomyelitis caused by lyssaviruses with a case fatality rate of almost 100%. Rabies virus (RABV) causes about 99% of all rabies cases in humans, mostly in Asia and Africa. Thirteen other lyssavirus species have been accepted by the International Committee on Taxonomy of Viruses [1] and two additional species have been identified Lleida bat lyssavirus from Spain in 2011 [2] and Gannoruwa Bat Lyssavirus from Sri Lanka in 2015 [3]. Bats are considered to be the true reservoir of lyssaviruses [4]. There is evidence that bats seroconvert after exposure to lyssaviruses without development of clinical signs, but in some cases, bats develop clinical disease similar to rabies in other mammals and death occurs after the appearance of clinical signs [5]. Finland has been free of RABV since 1991, but European bat lyssavirus type 2 (EBLV-2) was detected in 2009 in a Daubenton’s bat (*Myotis daubentonii*) [6]. In addition, antibodies against lyssavirus have been detected in Daubenton’s bats from the same area [7]. EBLV-2 has sporadically been isolated from bats in the Netherlands [8], Switzerland [9], the United Kingdom [10], Finland [6], Germany [11] and Norway [12]. EBLV-2 has caused two human cases: in Finland in 1985 [13] and in the United Kingdom in 2002 [14]. Both victims were researchers studying bats and they did not receive pre- or post-exposure rabies prophylaxis. No spill-over infections to other mammals than humans have been detected for EBLV-2.

**Fig. 1:**
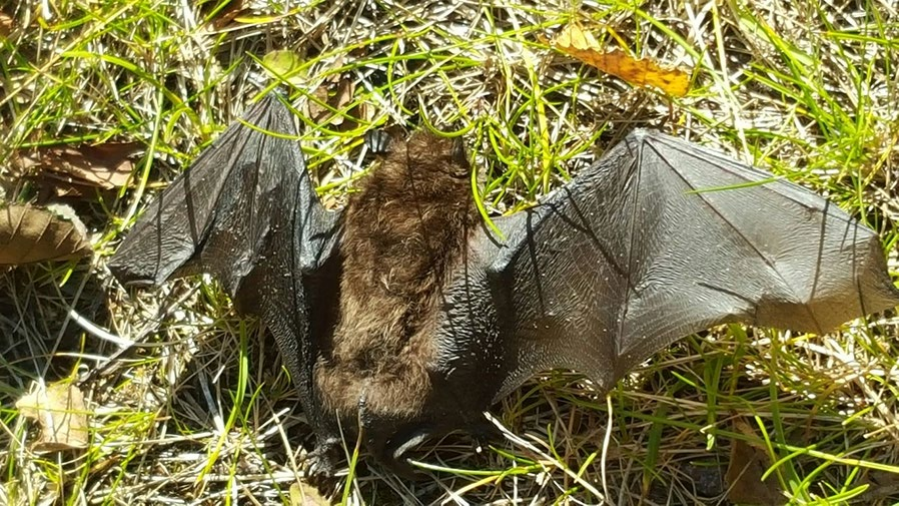
The Daubenton’s bat found dead in the municipality of Inkoo, SW Finland. Courtesy of Riitta Räisänen

A private citizen observed an abnormally behaving bat at a summer cottage in the municipality of Inkoo (60°02′45″N, 024°00′20″E) in October, 2016. Inkoo is in the province of southern Finland and is part of the Uusimaa region. The bat exhibited behavioral changes: it appeared during daytime, was unable to crawl into the roof space of a building, and had difficulties in moving and flying. When crawling on the wall, the bat had severe ataxia and tetraparesis. Later that day, the bat was grounded and found dead. It was sent to the Finnish Food Safety Authority Evira for autopsy. The Daubenton’s bat was a cachexic adult female, weighing circa 7 g (Fig. 1).

The presence of lyssavirus was detected in the brain by fluorescent antibody test (FAT) [15]. Smears prepared from a sample of brain tissue were fixed in high-grade cold acetone, air dried, and then stained with specific conjugate (FITC Anti-Rabies Monoclonal Globulin, Fujirebio Diagnostics and Rabies Antinucleocapsid conjugate, Bio-Rad). FAT slides were examined for specific fluorescence using a fluorescence microscope with positive results. The brain suspension of the bat was inoculated in mouse neuroblast cells Neuro-2a (ATCC^®^ CCL-131™) according to a rabies tissue culture infectious test (RTCIT) procedure described in the OIE manual [15], and the virus isolation for lyssavirus was positive. Additional organ and swab samples were collected from the bat. RNA was extracted from the organ and swab suspensions of the bat with the QIAamp Viral RNA Mini Kit (Qiagen, Hilden, Germany) according to the manufacturer’s instructions. The OneStep RT-PCR kit (Qiagen) was used to amplify two fragments. The reaction volume was 25 µL and the temperature profile of cDNA synthesis and amplification was 30 min at 50 °C, 15 min at 94 °C for reverse transcriptase inactivation and DNA polymerase activation, followed by 30 amplification cycles of 1 min at 94 °C, 1 min at 50 °C, and 1 min at 72 °C. Primers were published by Davis et al. [16]. The results are presented in Table 1. After agarose gel electrophoresis, the band of the brain sample was cut from the gel and DNA was extracted with the Qiaquick Gel Extraction Kit (Qiagen). The reaction products were purified using the DyeEx 2.0 Spin Kit (Qiagen). PCR products were sequenced using an ABI 3100 Avant Genetic Analyzer (Applied Biosystems) with the primers used in the PCR and a Big Dye Terminator v3.1 Cycle sequencing kit (Applied Biosystems). The sequences were analyzed with DNASTAR Lasergene 10.

**Table 1:** Results of lyssavirus detection from different organ and swab samples from the Daubenton’s bat

	Brain	Spinal cord	Salivary gland	Mouth swab	Anal swab	Tongue	Intestines	Liver	Eye	Lung	Trachea	Bladder	Tonsils
FAT	+	+	NA	NA	NA	NA	NA	NA	NA	NA	NA	NA	NA
RTCIT	+	+	+	−	−	−	−	−	−	−	−	−	−
RT-PCR	+	+	+	−	−	−	−	−	−	−	−	−	−

*FAT* fluorescent antibody test, *RTCIT* rabies tissue culture infectious test, *NA* not applicable

There have been two cases of EBLV-2 from Daubenton’s bats in Finland, the first in 2009 [6] and now in 2016. This provides further evidence that EBLV-2 is enzootic in Daubenton’s bats in Finland, at least in the southwestern part of the country. However, we consider the risk of EBLV-2 infection of humans as extremely low. Adequate information should be given to the general public on what to do when they come into contact with bats. People who handle bats due to their work or hobby should be vaccinated against rabies according to the guidelines of WHO [17].

Even though Daubenton’s bats are most likely the true reservoir of EBLV-2, they can become diseased and show typical neurological signs of rabies: abnormal behavior, paralysis, and coma followed by death. Therefore, passive surveillance of sick and dead bats is the most important surveillance method. Researchers studying bats and members of the public play a key role in providing samples to the diagnostics laboratory. Active surveillance of healthy bats has seldom revealed lyssaviruses from bats [7, 18].

FAT has been proved to be effective in detecting EBLV-2 from infected bats, but in one recorded case, the FAT test was negative on a bat sample even though viral RNA was detected by RT-PCR and the virus was isolated in a cell culture. Some laboratories have had difficulties to reliably detect EBLV strains when using the FAT, with results depending on the rabies virus antibody conjugate and even the batch used [12].

**Fig. 2:**
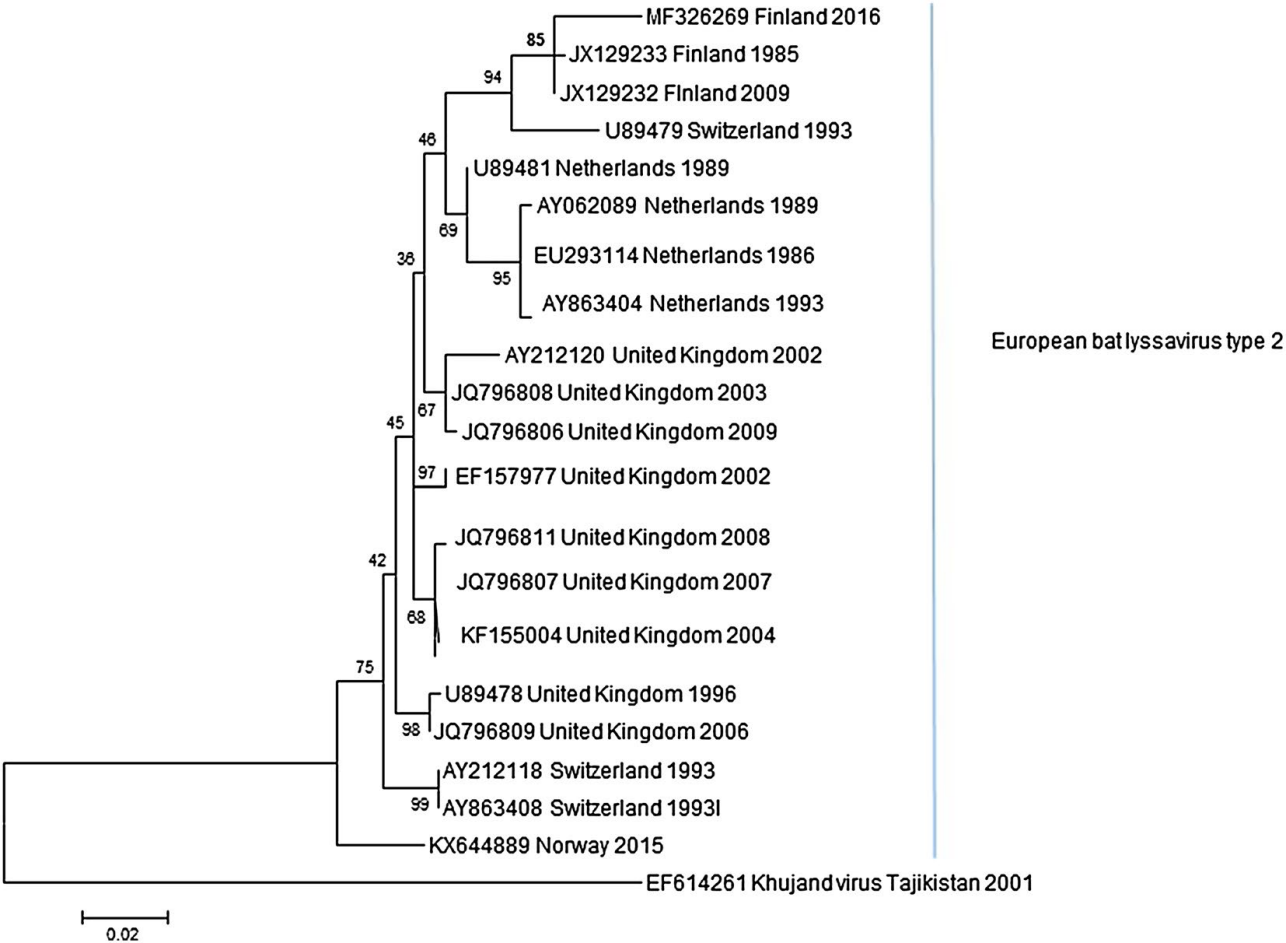
A phylogenetic tree based on partial N-gene sequences. The phylogenetic tree was estimated using the maximum likelihood approach in the program MEGA with 1000 bootstrap replicates

The viral RNA was detected by RT-PCR and the viable virus was isolated using mouse neuroblast cells from the brain, the spinal cord and the salivary glands, but not from other organ or swab samples (Table 1). The virus was identified as EBLV-2 based on partial N-gene sequencing and phylogenetic analysis (Fig. 2). The sequence (GenBank Accession Number MF326269) was 98% identical to previously found EBLV-2 strains in Finland. The isolated EBLV-2 was also phylogenetically very similar to the strains characterized in other parts of Europe [19]. Characterization of the isolated lyssaviruses provides valuable information on the epidemiological situation of the reservoir of specific lyssavirus and possible spill-over species.

## Abbreviations

EBLV-2: European bat lyssavirus type 2
FAT: fluorescent antibody test
OIE: World Organisation for Animal Health
RABV: rabies virus
RTCIT: rabies tissue culture infectious test
WHO: World Health Organization

## References

[1] International Committee on Taxonomy of Viruses. https://talk.ictvonline.org/taxonomy/. Accessed 10 June 2017.

[2] Arechiga-Ceballos NA, Morón SV, Berciano JM, Nicolás O, López CA, Juste J (2013). Novel Lyssavirus in Bat, Spain. Emerg Infect Dis..

[3] Gunawardena PS, Marston DA, Ellis RJ, Wise EL, Karawita AC, Breed AC (2016). Lyssavirus in Indian Flying Foxes, Sri Lanka. Emerg Infect Dis..

[4] Badrane H, Tordo N (2001). Host switching in Lyssavirus history from the Chiroptera to the Carnivora orders. J Virol.

[5] Banyard AC, Healy DM, Brookes SM, Voller K, Hicks DJ, Núñez A, Fooks AR (2014). Lyssavirus infection: ‘low dose, multiple exposure’ in the mouse model. Virus Res.

[6] Jakava-Viljanen M, Lilley T, Kyheröinen E-M, Huovilainen A (2010). First encounter of European bat lyssavirus type 2 (EBLV-2) in a bat in Finland. Epidemiol Infect.

[7] Nokireki T, Huovilainen A, Lilley T, Kyheröinen E-M, Ek-Kommonen C, Sihvonen L, Jakava-Viljanen M (2013). Bat rabies surveillance in Finland. BMC Vet Res.

[8] Van der Poel WHM, Van der Heide R, Verstraten E, Takumi K, Lina PHC, Kramps JA (2005). European bat lyssaviruses, the Netherlands. Emerg Infect Dis.

[9] Megali A, Yannic G, Zahno ML, Brügger D, Bertoni G, Christe P (2010). Surveillance for European bat lyssavirus in Swiss bats. Arch Virol.

[10] Whitby JE, Heaton PR, Black EM, Wooldridge M, McElhinney LM, Johnstone P (2000). First isolation of a rabies-related virus from a Daubenton’s bat in the United Kingdom. Vet Rec..

[11] Freuling C, Grossmann E, Conraths FJ, Schameitat A, Kliemt J, Auer E (2008). First isolation of EBLV-2 in Germany. Vet Microbiol.

[12] Moldal T, Vikøren T, Cliquet F, Marston DA, van der Kooij J, Madslien K, Ørpetveit I (2017). First detection of European bat lyssavirus type 2 (EBLV-2) in Norway. BMC Vet Res..

[13] Lumio J, Hillbom M, Roine R, Ketonen L, Haltia M, Valle M (1986). Human rabies of bat origin in Europe. Lancet.

[14] Fooks AR, McElhinney LM, Pounder DJ, Finnegan CJ, Mansfield K, Johnson N (2003). Case report: isolation of a European bat lyssavirus type 2a from a fatal human case of rabies encephalitis. J Med Virol.

[15] World Organization for Animal Health. Manual of Diagnostic Tests and Vaccines for Terrestrial Animals. http://www.oie.int/fileadmin/Home/eng/Health_standards/tahm/2.01.17_RABIES.pdf. Accessed 2 May 2016.

[16] Davis P, Holmes E, Larrous F, Van der Poel W, Tjørnehøj K, Alonso W (2005). Phylogeography, population dynamics and molecular evolution of European Bat Lyssaviruses. J Virol.

[17] Kessels JA, Recuenco S, Navarro-Vela AM, Deray R, Vigilato M, Ertl H (2017). Pre-exposure rabies prophylaxis: a systematic review. Bull World Health Organ.

[18] Schatz J, Fooks AR, McElhinney L, Horton D, Echevarria J, Vázquez-Moron S (2013). Bat rabies surveillance in Europe. Zoonoses Public Health.

[19] Jakava-Viljanen M, Nokireki T, Sironen T, Vapalahti O, Sihvonen L, Huovilainen A (2015). Evolutionary trends of European bat lyssavirus type 2 including genetic characterization of Finnish strains of human and bat origin 24 years apart. Arch Virol.

